# Supplementary material for: From sequence to enzyme mechanism using multi-label machine learning
Source: BMC Bioinformatics. 2014 May 19;15:150. doi: 10.1186/1471-2105-15-150 (PMC4229970; doi:10.1186/1471-2105-15-150)
Supplement: Additional file 2 — Java code of ml2db. Additional file ml2db_code.tar.gz contains the Java source code to run the multi-label machine learning experiments and save the results to database. The code’s Javadoc is included. [file 1471-2105-15-150-S2.zip › additional file 2/ml2db/ml2db/doc/main/ml2db/graphviz/GraphVizArff.html]

GraphVizArff


JavaScript is disabled on your browser.


- Overview
- Package
- Class
- Use
- Tree
- Deprecated
- Index
- Help

- Prev Class
- Next Class

- Frames
- No Frames

- All Classes

- Summary:
- Nested |
- Field |
- Constr |
- Method

- Detail:
- Field |
- Constr |
- Method


main.ml2db.graphviz

## Class GraphVizArff

- java.lang.Object
- - main.ml2db.graphviz.GraphViz
  - - main.ml2db.graphviz.GraphVizArff

- Direct Known Subclasses:
  :   GraphVizArffColoured

  ---

    

  ```
  public class GraphVizArff
  extends GraphViz
  ```

  Generates a GraphViz representation (.dot file) of the instances, class
  labels and attributes in a Weka/Mulan dataset and their relations. An image
  can be created from the .dot file.

  Version:
  :   14 Apr 2012

  Author:
  :   Luna De Ferrari ldf(at)st-andrews.ac.uk or
      luna.deferrari@st-andrews.ac.uk

- - ### Field Summary

    Fields

    | Modifier and Type | Field and Description |
    | --- | --- |
    | `static java.lang.String` | `BASE_ATTRIBUTE_SHAPE` |
    | `static java.lang.String` | `BASE_CLASS_SHAPE` |
    | `static java.lang.String` | `BASE_INSTANCE_SHAPE` |

    - ### Fields inherited from class main.ml2db.graphviz.GraphViz

      `DOT, DOT_COMMENT_PREFIX, DOT_UNDIRECTED_EDGE, FIG, GIF, JPG, PDF, PLAIN, PNG, PS, SVG`
  - ### Constructor Summary

    Constructors

    Constructor and Description

    `GraphVizArff(java.lang.String title)`
  - ### Method Summary

    Methods

    | Modifier and Type | Method and Description |
    | --- | --- |
    | `void` | `addInstances(IdMultiLabelInstances instances)` |
    | `java.lang.String` | `completeDotLine(java.lang.String line)` |
    | `void` | `fillDot(IdMultiLabelInstances instances)` |
    | `java.lang.String` | `getBaseDotAllInstances()` |

    - ### Methods inherited from class main.ml2db.graphviz.GraphViz

      `add, addln, addln, end_graph, getDotSource, getGraphImage, readSource, start_graph, toGif, toGifToTmp, toImage, toImageFile, toJpg, toPdf, writeDotSourceToFile, writeGraphToFile, writeGraphToFile`
    - ### Methods inherited from class java.lang.Object

      `equals, getClass, hashCode, notify, notifyAll, toString, wait, wait, wait`

- - ### Field Detail


    - #### BASE\_INSTANCE\_SHAPE

      ```
      public static final java.lang.String BASE_INSTANCE_SHAPE
      ```

      See Also:
      :   Constant Field Values


    - #### BASE\_CLASS\_SHAPE

      ```
      public static final java.lang.String BASE_CLASS_SHAPE
      ```

      See Also:
      :   Constant Field Values


    - #### BASE\_ATTRIBUTE\_SHAPE

      ```
      public static final java.lang.String BASE_ATTRIBUTE_SHAPE
      ```

      See Also:
      :   Constant Field Values
  - ### Constructor Detail


    - #### GraphVizArff

      ```
      public GraphVizArff(java.lang.String title)
      ```
  - ### Method Detail


    - #### addInstances

      ```
      public void addInstances(IdMultiLabelInstances instances)
      ```


    - #### completeDotLine

      ```
      public java.lang.String completeDotLine(java.lang.String line)
      ```


    - #### fillDot

      ```
      public void fillDot(IdMultiLabelInstances instances)
      ```


    - #### getBaseDotAllInstances

      ```
      public java.lang.String getBaseDotAllInstances()
      ```

      Returns:
      :   m\_baseDotAllInstances


- Overview
- Package
- Class
- Use
- Tree
- Deprecated
- Index
- Help

- Prev Class
- Next Class

- Frames
- No Frames

- All Classes

- Summary:
- Nested |
- Field |
- Constr |
- Method

- Detail:
- Field |
- Constr |
- Method
